# Supplementary material for: Selection and Comparative Gene Expression of Midgut-Specific Targets for Drosophila suzukii
Source: Insects. 2023 Jan 12;14(1):76. doi: 10.3390/insects14010076 (PMC9864236; doi:10.3390/insects14010076)
Supplement: Supplementary file 1 [file insects-14-00076-s001.zip › Table S1-S4.pdf]

Table S1. Raw data of the midgut and whole-body transcriptome results.

Sample	Total read base	Total reads	GC (%)	Q (20)	Q (30)
Midgut	5,497,308,800	54,428,800	52.49	98.13	96.79
Whole-body	6,040,056,944	59,802,544	52.57	98.11	96.78

Total read bases: Total reads x Read length; Total reads: Total number of reads; GC(%): GC content; Q20 (%): Ratio of reads that have phred quality score over 20; Q30 (%): Ratio of reads that have phred quality score over 30.

Table S2. Mapped data stats. # of processed reads: # of cleaned reads after trimming. Number of mapped reads: # of reads mapped to reference. # of suppressed reads by multiple mapping: Number of reads removed due to multiple mapping. overall read mapping ratio: Number of total mapped reads / # of total processed reads.

Sample	Read type	# of processed reads	# of mapped reads	# of suppressed reads by multiple mapping	Overall read mapping ratio
**Midgut**	1	27,105,105	21,403,526 (79.0%)	1,507,409 (7.0%)	78.9%
**Midgut**	2	27,105,105	21,363,944 (78.8%)	1,505,599 (7.0%)	
**Whole-body**	1	29,777,714	23,486.620 (78.9%)	1,601,068 (6.8%)	78.8%
**Whole-body**	2	29,777,714	23,429,654 (78.7%)	1,593,793 (6.8%)	

Table S3. The most upregulated genes in the midgut of *Drosophila suzukii* are found in *Drosophila melanogaster*.

Search Gene	Found FBGN	Found Gene	imaginal disc, larvae L3	central nervous system, larvae L3	head, virgin 1-day female	salivary gland, larvae L3	digestive system, larvae L3	digestive system, 1-day adult	digestive system, 4-day adult	digestive system, 20-day adult	fat body, larvae L3	carcass, larvae L3	ovary, mated 4-day female	testis, mated 4-day male
**CG3934**	FBgn0037783	**Npc2c**	19	0	0	2	155	25	8	10	0	1	0	0
**CG7170**	FBgn0035887	**Jon66Cii**	302	0	0	43	1430	5	2	11	1	5	0	0
**CG18548**	FBgn0042206	**GstD10**	6	0	1	1	14	54	33	20	68	1	0	7
**CG5246**	FBgn0038484	**CG5246**	9	0	0	1	42	66	38	38	1	0	0	2
**CG14746**	FBgn0043576	**CG14743**	81	0	0	2	147	302	227	200	12	1	0	3
**CG7118**	FBgn0035886	**Jon66Ci**	1519	0	0	318	8900	43	27	116	2	32	0	0
**CG12092**	FBgn0261675	**Npc1b**	1	0	0	0	18	41	36	43	0	0	0	0
**CG8221**	FBgn0020506	**Amyrel**	28	0	0	1	36	6	2	4	0	0	0	0
**CG8695**	FBgn0002571	**Mal-A3**	26	0	0	0	20	200	88	112	0	0	0	14
**CG7381**	FBgn0038098	**CG7381**	3	1	4	0	46	74	71	80	0	1	21	1
**CG30502**	FBgn0050502	**fa2h**	1	0	0	0	11	34	34	37	0	0	0	0
**CG14820**	FBgn0035718	**CG14820**	1	0	0	0	8	64	45	32	0	0	0	1
**CG33346**	FBgn0053346	**CG33346**	25	2	0	11	833	341	175	216	1	5	0	2
**CG10827**	FBgn0038845	**CG10827**	24	0	0	1	84	233	131	166	0	0	0	10
**CG40305**	FBgn0044872	**FucTC**	0	0	0	0	1	3	2	2	0	0	0	0
**CG9903**	FBgn0030756	**CG9903**	9	0	0	6	241	22	12	12	0	1	0	1
**CG15818**	FBgn0031910	**CG15818**	59	10	0	26	2001	53	47	60	0	15	0	0
**CG8907**	FBgn0038466	**CG8907**	5	0	0	1	35	69	55	79	1	0	0	1
**CG6417**	FBgn0032435	**Oatp33Eb**	6	0	0	1	57	55	46	69	0	0	0	2

Table S4. List of the primers used in this study

Gene Name	DS Number	Forward (5' - 3')	Reverse (5' - 3')
**DH31-R**	**DS10_00006592**	GGATTCGACCCAGCAAGATT	TTACGCAGGTGGTGTAGTTG
**Toll-9**	**DS10_00007171**	GCTCTTCATGTCGGGCAATA	GTAGGTCAGGAAGTTGGTGAAG
**snakeskin**	**DS10_00008095**	ACGTGGTTGGCTGCATTA	CTCCGAGTTCACATAGAGGAATC
**methuselah**	**DS10_00013361**	GTTCCCGAATGGTTCGTATCT	CTTATGTGGCTCTCCACCTTT
**NPF-R**	**DS10_00008044**	GTGAGTCAGGCCACTCTTT	GACATGCTCTGGGAGTTCTT
**Gr43a**	**DS10_00005654**	AGACCATGTACCGCATCAAG	TTCTCGTAGCCCAGGAAGTA
**Adsn1-R**	**DS10_00012673**	CGGAGGAATCCACTGAAACTAA	CTGATCGTGGTTGCTGACTAT
**Adsn2-R**	**DS10_00012139**	GCCGCCGATGTCTTTAGTAT	TCCGTGGTCATGACTTTCTG
**Rpn2**	**Reference 1**	TCAGCGGCAACTTCTCCTTT	CGCGAGATATTCAGCTCCTCA
**Rpt6**	**Reference 2**	AGAAGGTGCTGGTCAAGGTG	TAGCTCTCATTTCGCAGGGC
